# The Use of Growth Factors and Other Humoral Agents to Accelerate and Enhance Burn Wound Healing

**Published:** 2011-11-07

**Authors:** Yiu-Hei Ching, Thomas L. Sutton, Yvonne N. Pierpont, Martin C. Robson, Wyatt G. Payne

**Affiliations:** ^a^Institute for Tissue Regeneration, Repair, & Rehabilitation Bay Pines VA Health Care System, Bay Pines, FL; ^b^Division of Plastic Surgery University of South Florida, Tampa

## Abstract

**Objective:** Certain cytokines, especially those known as growth factors, have been demonstrated to mediate or modulate burn wound healing. Experimental and clinical evidence suggests that there are therapeutic advantages to the wound healing process when these agents are utilized. Positive effects have been reported for 4 types of wounds seen in the burn patient: partial-thickness wounds, full-thickness wounds, interstices of meshed skin grafts, and skin graft donor sites. **Methods:** A comprehensive literature search was performed using the MEDLINE, Ovid, and Web of Science databases to identify pertinent articles regarding growth factors and other cytokines in burns and wound healing. **Results:** The current knowledge about cytokine growth factors and their potential therapeutic applications in burn wound healing are discussed and reviewed. **Conclusions:** Platelet-derived growth factor, fibroblast growth factors, epidermal growth factors, transforming growth factor alpha, vascular endothelial growth factor, insulin-like growth factor I, nerve growth factor, transforming growth factor beta, granulocyte-macrophage colony-stimulating factor, and amnion-derived cellular cytokine solution have all been suggested to enhance the rate and quality of healing in 1 or more of these wounds encountered in burn care.

The isolation and investigation of a class of regulatory polypeptides known as cytokine growth factors and their role in wound healing began almost 30 years ago. However, their potential as therapeutic agents in acute wound healing has yet to be realized, despite experimentally and clinically demonstrated safety and efficacy and extensive experience in the treatment of chronic wounds.^1^^-^6 Burn wounds represent the largest of routinely cared-for wounds, and they remain a significant clinical problem for both caregivers and patients. The promise of utilizing growth factors and other humoral agents in accelerating and enhancing wound healing represents an important goal in burn wound care. This article reviews the current literature regarding the use of platelet-derived growth factor (PDGF), fibroblast growth factors (FGB), epidermal growth factors (EGF), transforming growth factor alpha (TGF-α), vascular endothelial growth factor (VEGF), insulin-like growth factor I (IGF-I), nerve growth factor (NGF), transforming growth factor beta (TGF-β), granulocyte-macrophage colony-stimulating factor (GM-CSF), and amnion-derived cellular cytokine solution (ACCS) as local and systemic agents in healing partial-thickness burn wounds, full-thickness burn wounds, interstices of meshed skin grafts, and skin graft donor sites.

## GROWTH FACTORS AND THEIR IMPLICATIONS IN WOUND HEALING

In the scope of wound healing, cytokine growth factors act to influence many processes including the proliferation and migration of various cell types, chemotaxis of fibroblasts and inflammatory cells, endothelial cell stimulation and angiogenesis, production and remodeling of extracellular matrix, inhibiting apoptosis, and mediating the synthesis of other cytokines and growth factors.^3^^,^^5^^,^^7^ The orderly, sequential progression through the stages of wound healing requires a number of different cells, humoral factors, and other agents to interact in such a way that timely and proper closure of the wound occurs. Aberrations in any of these pathways may result in pathologic wound healing from chronic, nonhealing wounds to keloid or hypertrophic scar formation.^7^ To this point, growth factors have been implicated in mediating the normal healing process as well as playing a role in impaired wound healing. For example, certain growth factors (PDGF and IGF-I) act to inhibit the apoptosis pathways necessary for the rapid turnover of cells required to facilitate the different stages of physiologic wound healing.^7^ The positive and negative effects of growth factors in wound healing must, therefore, be taken into consideration for therapeutic applications. While there is a theoretical concern for oncogenesis secondary to growth factor therapy, tumor development has not been seen in clinical or experimental studies to our knowledge.

Platelet-derived growth factor is a key mediator in wound healing, and its importance is highlighted in that it was the first recombinant growth factor approved for topical application to accelerate wound closure.^1^^,^^8^ Platelet-derived growth factor is released by platelets and secreted by activated macrophages with concentrations greatest in the early stages of the injury response, peaking by day 3.^9^^-^13 However, PDGF plays a role throughout the entire process of wound healing. In the beginning, PDGF serves as a chemoattractant for the migration of fibroblasts, neutrophils, and monocytes to the site of injury.^3^^,^^14^ Platelet-derived growth factor is also a mitogen for fibroblasts and promotes the production of new extracellular matrix components.^14^^,^^15^ Later in the proliferative phase, PDGF stimulates the differentiation of fibroblasts into myofibroblasts, promoting contraction of collagen matrices and the wound.^14^^-^16 Platelet-derived growth factor has also been implicated in the remodeling phase through its effect on stimulating collagenase production in fibroblasts.^16^ Of the 3 isoforms of PDGF identified, PDGF-BB has been found to be the most beneficial for wound healing.^17^^,^^18^

Fibroblast growth factors constitute a large family of mitogens affecting a multitude of cell types with important implications for wound healing.^8^^,^^14^^,^^19^ In wounds, expression of FGF detected by immunohistochemical methods is greatest at the center with progressive decline in intensity toward the periphery and almost none found in uninjured skin.^20^ Acidic fibroblast growth factor (aFGF) and basic fibroblast growth factor (bFGF) have been identified in wound fluid, particularly at the earliest stages of healing.^8^^,^^9^^,^^13^^,^^20^^-^25 Basic FGF was further demonstrated to derive from fibroblasts in cultured skin substitutes (CSS).^26^ The role of bFGF in wound healing has been shown in vitro and in vivo through its actions on early local macrophage activation, augmenting production of extracellular matrix components, proliferation and differentiation of neuroectodermal and mesodermal derivatives, fibroblast proliferation, endothelial cell proliferation and migration for angiogenesis, and reepithelialization.^3^^,^^24^^,^^27^^-^30 The broadest experience with clinical trials of growth factor therapy in wound healing has been with bFGF.^8^ Acidic FGF, similar in its biologic properties to bFGF, likewise influences the proliferation of fibroblasts and endothelial cells, promoting wound healing and angiogenesis.^31^^,^^32^ Keratinocyte growth factor 1 (KGF-1), another member of the FGF family, promotes reepithelialization through its effects on the proliferation and differentiation of epithelial cells while inhibiting apoptosis.^33^^,^^34^ Keratinocyte growth factor 2 (KGF-2) similarly affects reepithelialization but also stimulates granulation tissue formation and collagen deposition in the healing wound.^8^^,^^33^^,^^35^ Further evidence suggests that early induction of KGF expression may be of importance for the rapid reepithelialization seen in normal wound healing.^36^

Epidermal growth factors comprise another family of mitogens found to be present in wound fluid and have significant effects on wound healing.^14^^,^^37^^-^40 Epidermal growth factor is mitogenic to epithelial, mesothelial, and endothelial cells, and it has been shown to accelerate reepithelialization, increase proliferation and tensile strength of healed dermis, and improve wound healing overall.^3^^,^^38^^,^^39^^,^^41^ Regarding burn wound healing, however, there is conflicting evidence about the presence of EGF in the wound fluid.^8^^,^^14^ Two other members of the EGF family, heparin-binding epidermal growth factor (HB-EGF) and TGF-α, have been detected at significant levels in burn wound fluid.^14^^,^^42^^,^^43^ Evidence for the roles of HB-EGF and TGF-α in wound healing is seen through their effects on stimulating keratinocyte proliferation and accelerating reepithelialization.^37^^,^^42^ Heparin-binding epidermal growth factor is also a mitogen for fibroblasts and acts to stimulate granulation tissue formation.^14^^,^^43^

Vascular endothelial growth factors include a family of cytokine growth factors identified as important mediators of angiogenesis, lymphangiogenesis, and vascular permeability.^44^^-^49 Recent evidence suggests that the proangiogenic effects of VEGF may be mediated through the stimulation of hydrogen sulfide synthesis from endothelial cells, in turn promoting their proliferation, migration, and permeability.^46^^,^^49^^,^^50^ There are also data to support the role of VEGF in stimulating epithelialization and collagen deposition.^51^ After injury, the expression of VEGF from wounded cells is significantly increased as part of the physiologic response, and the same effect can be induced experimentally from quiescent cells through direct exposure to serum, EGF, KGF, TGF-β1, and tumor necrosis factor alpha.^52^ Several studies have demonstrated the benefit of exogenous VEGF therapy on accelerating and enhancing wound healing through topical application, amplified expression, and adenovirus-vector gene transfer of the growth factor.^53^^-^56 Conversely, neutralizing antibodies to VEGF-A significantly decreased wound fluid accumulation, granulation tissue formation, and angiogenesis, further evidence for the role of VEGF (VEGF-A, in particular) in wound healing.^46^^,^^57^ Two other VEGF family members, VEGF-C and VEGF-D, are involved in regulating lymphangiogenesis but not angiogenesis.^14^^,^^58^ The importance of lymphatic vasculature in wound healing is demonstrated through its presence in formed granulation tissue and insufficiency in chronic wounds with impaired healing.^45^^,^^58^ While VEGF appears to be beneficial in promoting burn wound healing, a caveat to this is evidence suggesting that the increased serum level of VEGF is responsible for the edema, anasarca, and edema-associated complications after burn injury.^46^

Insulin-like growth factors I and II are well-described proanabolic and anticatabolic agents that act on a multitude of cell types systemically.^14^^,^^59^ The positive anabolic and wound healing effects of growth hormone (GH) appear to be directly and indirectly associated IGF-1 expression.^3^^,^^59^^-^66 In the scope of wound healing, evidence suggests that IGF-I is mitogenic for keratinocytes and fibroblasts, inhibits apoptosis pathways, attenuates pro- and anti-inflammatory cytokine production, and stimulates extracellular matrix component production.^67^^-^70 IGF-I is found in burn wound fluid as part of the normal injury response, and the expression of IGF-I and IGF-II has been shown to be significantly upregulated in epidermal cells of healing wounds that normally express minimal amounts of the protein product.^14^^,^^71^^,^^72^ In addition, the levels of IGF-I in wound fluid demonstrate a positive correlation with the success of wound healing.^73^

Nerve growth factor has been demonstrated to affect multiple aspects of wound healing.^14^^,^^74^ Given the variety of neurogenically mediated processes involved in wound healing, cutaneous nerve growth and restoration of normal innervation regulated by NGF are essential to the normal healing process.^74^^,^^75^ Sensory nerves are responsible for releasing substance P, a neuropeptide associated with stimulating epithelial proliferation and migration and accelerating wound healing in a murine model when applied topically.^75^^-^77 Nerve growth factor has also been found to have proliferative and antiapoptotic effects on keratinocytes and endothelial cells and to promote fibroblast migration.^78^^-^80 Through its proinflammatory effects on neutrophils, eosinophils, mast cells, and T lymphocytes, NGF may serve to enhance the injury response as well.^79^

The TGF-β family is composed of many members with a great diversity in their actions.^14^^,^^81^ Of particular interest to wound healing, the TGF-β1, TGF-β2, and TGF-β3 isoforms have been found to share a number of functions such as stimulating the proliferation and production of fibroblasts, extracellular matrix components, and cell adhesion molecules, while inhibiting that of keratinocytes and most other cells.^3^^,^^8^^,^^82^^-^86 Early evidence on TGF-β suggested that these factors were involved in accelerating reepithelialization and enhancing wound healing. In a rodent partial-thickness injury model, TGF-β1 expression was observed to share a close spatial and temporal correlation with the progression of wound healing.^87^ TGF-β1 was also found to promote keratinocyte migration and epithelial regeneration through enhancing integrin production and binding of keratinocytes to fibronectin and vitronectin.^84^^,^^85^^,^^88^ This suggested an overall positive effect on reepithelialization despite the antiproliferative effects of TGF-β1 on keratinocytes.^88^ Accordingly, TGF-β1-deficient mice were found to have poor wound healing with impaired granulation tissue formation, extracellular matrix production, and reepithelialization.^89^ Interestingly, another study identified that TGF-β1 deficiency was associated with delays in wound healing only if TGF-β2 and TGF-β3 were not expressed.^81^ However, there is recent evidence suggesting that TGF-β1 and TGF-β2 negatively influence wound healing through retarding reepithelialization and increasing scar formation and contraction.^90^^,^^91^ TGF-β1 and TGF-β2 have been implicated in keloid development after discovery of their overexpression in these tissues.^92^^,^^93^ As TGF-β also serves as an anti-inflammatory agent, any state of persistent or increased inflammation such as infection or protracted healing may lead to overexpression of TGF-β and subsequent keloid or hypertrophic scar formation.^7^ On the contrary, fetal wounds that are less prone to cutaneous scarring are associated with low levels of TGF-β1 and TGF-β2.^14^^,^^94^^,^^95^ These findings are further supported by evidence from studies on TGF-β1 and TGF-β2 antagonism.^90^ Intradermal injection of neutralizing antibodies to TGF-β1 alone or TGF-β1 and TGF-β2 was associated with reduced scarring and wound contraction.^96^^-^98 The use of a synthetic TGF-β peptagonist applied locally was found to accelerate reepithelialization, enhance overall wound healing, and decrease scar formation and contracture in rabbit excisional and porcine burn and excisional wound models.^91^^,^^99^ Contrary to the negative effects of TGF-β1 and TGF-β2, TGF-β3 appears to be beneficial for improving wound healing.^95^ The expression of TGF-β3 is high in fetal wounds compared to adult wounds that are more prone to scarring.^94^ Experimental models of TGF-β3 deficiency and deletion demonstrated delayed wound healing and increased scar formation.^95^^,^^100^ Conversely, the therapeutic use of recombinant human TGF-β3 (Avotermin, Juvista, Renovo Group, Manchester, United Kingdom) has been found to accelerate wound healing and improve the appearance of cutaneous scarring postoperatively.^95^

Granulocyte-macrophage colony-stimulating factor is a multipotential cytokine with many diverse functions.^101^^-^103 In the scope of burn injury, the benefit of GM-CSF is 2-fold. It has positive effects on enhancing wound healing and also acts to promote and restore an impaired immune response.^103^ In normal circumstances, GM-CSF is only minimally expressed by keratinocytes.^104^^,^^105^ However, significant upregulation and production of this cytokine from keratinocytes, fibroblasts, endothelial cells, macrophages, and dendritic cells occur shortly after tissue injury as part of the wound healing process.^8^^,^^104^^-^110 Granulocyte-macrophage colony-stimulating factor has been shown to accelerate reepithelialization, promote neovascularization, and improve the quality and quantity of granulation tissue formation through increasing keratinocyte and endothelial cell proliferation, migration, and survival.^101^^,^^102^^,^^106^^,^^107^^,^^111^^,^^112^ While GM-CSF is also known to affect fibroblasts, its role in tissue remodeling is complex, and there is conflicting evidence about its benefit.^102^^,^^113^^-^116 Granulocyte-macrophage colony-stimulating factor plays an important part in mediating the production and release of other cytokines and growth factors as well.^101^^,^^102^ The stimulation of interferon-γ, interleukin-6, and TGF-β1 has demonstrated value in accelerating and enhancing wound healing.^101^ Decreased synthesis of IL-2, as well as impaired T cell and granulocyte proliferation and function, has been associated with thermal injury leading to a weakened immune response.^117^^-^121 Granulocyte-macrophage colony-stimulating factor has been shown to restore these immune deficits, suggesting an additional role for its use in burns to decrease morbidity and mortality from infectious complications.^121^^,^^122^

To this point, the discussion has focused upon growth factors and cytokines and their applications in wound healing on an individual level. The normal wound healing process, however, involves a complex series of interactions among a diverse population of cell types, growth factors, cytokines, and other mediators in a sequential fashion.^3^^,^^67^^,^^123^^,^^124^ There is evidence to suggest that the use of combination or sequential cytokine agent therapy is beneficial for wound healing.^125^ One such combination is the autologous platelet product of platelet-derived wound healing factors (PDWHF) as reported by Knighton et al.^126^^,^^127^ Platelet-derived wound healing factor is prepared from the isolation of platelet α-granule products from platelet-rich plasma separated from patient whole blood.^126^ Administered as a topical salve, PDWHF was found to be efficacious in promoting the healing of chronic nonhealing wounds secondary to a number of disease processes.^126^ However, there are limitations to PDWHF including the need for dilution in buffered saline and the requirement for supraphysiologic concentrations of growth factors and cytokines required for therapeutic effect.^124^^,^^126^ Another combination therapy, ACCS, has been studied experimentally and demonstrated to be valuable in several wound healing applications.^125^^,^^128^^-^130 Amnion-derived cellular cytokine solution is the cytokine-rich product secreted from amnion-derived multipotent progenitor (AMP) cells.^124^^,^^131^ Initially, the use of AMP cells on laparotomy wound healing in a rodent model was found to increase the gain of incisional breaking strength and decrease the incidence and severity of acute wound failure.^131^ The production of ACCS from these AMP cells was thought to be one possible mechanism of this benefit. Amnion-derived cellular cytokine solution is composed of a number of naturally appearing growth factors and cytokines involved in normal wound healing at physiologic concentrations, including PDGF, VEGF, TGF-β2, angiogenin, tissue inhibitor of metalloproteinase-1 (TIMP-1) and -2 (TIMP-2).^124^^,^^129^ Angiogenin plays an important role in angiogenesis through activation of endothelial and smooth muscle cells to stimulate their proliferation, migration, and invasion.^132^ TIMP-1 and TIMP-2 are particularly beneficial for wound healing through their effects on promoting cell proliferation, preventing apoptosis, and inhibiting matrix metalloproteinases (MMPs) that have been implicated in impeding wound healing directly by destroying extracellular matrix proteins and indirectly through degradation of growth factors and cytokines.^8^^,^^90^^,^^124^^,^^133^ This is particularly pertinent in burn wounds associated with increased tissue proteolysis secondary to significant upregulation of MMP-1, MMP-3, and MMP-9 while TIMP-1 and TIMP-2 are relatively unaffected.^134^^,^^135^ Other combination growth factors demonstrated to be of value in experimental wound healing models include PDGF and TGF-α, PDGF and IGF, bFGF and IGF, PDGF and FGF, and KGF- and IGF-1-cDNA transferred together.^136^^-^142 Live yeast cell derivative, an alcohol extract from *Saccharomyces cerevisiae*, has also been demonstrated to improve burn and other types of wound healing.^143^^-^145 However, the peptide portion of live yeast cell derivative implicated in wound healing does not appear to contain any of the described growth factors and is significantly less effective by comparison.^143^ The use of sequential cytokine therapy has also been examined in chronic wounds using GM-CSF and bFGF therapy, but the results of this study suggested that bFGF alone was more beneficial than sequential therapy.^146^

## PARTIAL-THICKNESS BURN WOUNDS

Partial-thickness burn wounds are defined as thermal injuries involving the epidermis and variable thicknesses of dermis. Superficial partial-thickness burn wounds are characterized by injury limited to the upper third of the dermis, leaving a sufficient blood supply to the wound bed to facilitate proper wound healing.^147^ Deep partial-thickness burn wounds, involving more of the dermal layer and a possible compromised blood supply, often give rise to complications such as delayed wound healing, aberrant scarring, and contracture formation.^147^ Growth factors and cytokines may be of therapeutic value with these types of injuries to accelerate and enhance healing.^90^

Of the 4 types of burn wounds discussed in this article, partial-thickness burn wounds have been the most extensively studied. In a prospective, randomized, placebo-controlled trial, Fu et al^148^ showed that the use of recombinant bovine bFGF on superficial and deep second-degree burn wounds improved healing time and quality. The time to healing was shortened from 12.4 to 9.9 days (*P* = .0008) and from 21.2 to 17.0 days (*P* = .0003) for superficial and deep second-degree burns, respectively.^148^ Histologic evaluation of these wounds revealed quantitative and qualitative improvements in cellular differentiation, angiogenesis, reepithelialization, and dermal-epidermal organization.^148^ Another prospective, randomized, placebo-controlled, double-blinded study examining topical recombinant aFGF on deep partial-thickness burn injuries (and skin graft donor sites, discussed later) demonstrated significantly accelerated healing compared to placebo.^31^ Ma et al^31^ noted that a higher percentage of wounds (71.79% vs 53.85%) were fully healed by 21 days postburn, and the mean healing time was shorter (17.23 vs 18.92 days, *P* = .035). Akita et al^149^ found improved functional and aesthetic outcomes of burn wound scars with the use of bFGF spray (Trafermin, Fiblast, Kaken Pharmaceutical, Tokyo, Japan) in pediatric patients with superficial and deep dermal burns. The function of the healed stratum corneum was assessed using a moisture meter (ASA-M2, Asahi Biomed, Co Ltd, Yokohama, Japan) that revealed decreased transepidermal water loss, increased epidermal water content, and increased thickness of the layer.^149^ Clinical evaluation of scar quality using the Vancouver Scar Scale based on pigmentation, pliability, height, and vascularity was significantly improved with bFGF therapy.^149^ A similar study by the same group examined the use of bFGF spray and burn wound healing in adults with second-degree burns.^30^ Treatment with bFGF again resulted in improved functional and aesthetic outcomes from the methods described earlier as well as Cutometer and durometer measurements of scar elasticity and hardness, respectively.^30^ There are other clinical trials in the literature describing the beneficial effects of bFGF on partial-thickness burn wound healing. One study demonstrated that treatment of cells in a healing wound with gene gun-mediated delivery of the human bFGF gene shortened complete healing time by 1.75 days (*P* < .05).^150^ Nie et al^151^ showed that topical oxygen therapy supplementing bFGF application accelerated deep second-degree burn wound healing. In addition, the use of bovine amnion in conjunction with topical recombinant bovine bFGF was found to decrease healing time for deep partial-thickness burn wounds.^152^

In 1986, Brown et al^153^ demonstrated that the use of topically applied EGF accelerated epidermal regeneration in porcine models of split-thickness wounds and partial-thickness burns. With the use of biosynthetic human EGF in silver sulfadiazine, complete epidermal regeneration was evidenced by day 7 while very little was seen in untreated burns or those treated with silver sulfadiazine alone.^153^ Cribbs et al^37^ showed that topical application of recombinant heparin-binding EGF in cholesterol-lecithin pellets significantly increased keratinocyte proliferation, enhanced endogenous TGF-α production, and accelerated reepithelialization in the decreasing mean wound area from 6.0 cm^2^ to 1.07 cm^2^ in treated mice compared with 2.20 cm^2^ in controls (*P* = .04) by postburn day 5. In a study by Jahovic et al,^40^ EGF was implicated as an important factor in the improved wound healing associated with saliva and wound licking. Epithelial disruption, dermal edema, follicular destruction, and myeloperoxidase activity (an indicator of neutrophil-mediated impairment of wound healing) were similarly and significantly decreased in rodent burn wounds treated with EGF alone and EGF combined with permitted wound licking.^40^ The beneficial effects of exogenous EGF were further demonstrated by Alemdaroğlu et al^39^ in a study comparing chitosan gel with and without EGF supplementation in rodent deep second-degree burn wounds compared to EGF solution alone, silver sulfadiazine alone, and no treatment. In this comparison, wound healing was improved in the EGF-supplemented chitosan gel and EGF-alone groups, with the most benefit seen in the former.^39^ On histologic assessment at day 14 postburn, the degree of cellular proliferation, epidermal thickness, and fibroblast maturation and differentiation were significantly better in these study groups, reflecting the effect of EGF in accelerating and enhancing burn wound healing.^39^ In a related study by the same group, EGF-containing liposomes were found to increase the rate of wound healing by the same measures described previously.^38^ Guo et al^154^ demonstrated that application of recombinant EGF on elderly burn patients decreased wound healing time from 16.22 to 14.3 days and from 29.13 to 26.11 days (*P* < .05) in superficial and deep second-degree burns, respectively. In addition, the use of an EGF-impregnated collagen sponge dressing for deep second-degree burns on a rabbit ear model was shown to enhance dermal matrix formation, wound breaking strength, and skin resilience, and decrease the rate of wound contraction.^155^

Evidence for VEGF as a valuable agent in partial-thickness burn wound healing comes from a study by Galeano et al.^55^ Through the use of a recombinant adeno-associated viral vector-mediated human VEGF (rAAV-VEGF165) gene transfer mechanism, injection of the rAAV-VEGF165 construct into the wounds of mice was found to enhance VEGF expression with an associated increase in epidermal and dermal proliferation and regeneration, angiogenesis, fibroblast proliferation, and maturation of the extracellular matrix.^55^ Erythropoietin, which interacts with VEGF and stimulates endothelial cell proliferation and migration, may also have a role as a wound healing agent.^156^ The same group demonstrated subcutaneous administration of recombinant human erythropoietin (rHuEPO) in mice increased epithelial proliferation, angiogenesis, extracellular matrix formation, and VEGF levels locally.^156^ This was associated with significantly increased reepithelialization and accelerated time to complete wound closure in 19 days (vs 23 and 24 days in controls and anti-rHuEPO-treated mice, respectively).^156^

The use of a TGF-β antagonist has been shown to positively affect partial-thickness burn wound healing.^91^ Singer et al^91^ demonstrated that treatment with a topical TGF-β peptagonist compared to carboxy methylcellulose, its vehicle, resulted in accelerated reepithelialization (90% vs 45% completely reepithelialized at 14 days, *P* = .002) and reduced scar formation and contraction in a porcine model. The proportion of contracted wounds at 28 days was significantly lower with TGF-β antagonist use (35% vs 65%, *P* = .02).^91^ An adjunct to the consideration of TGF-β manipulation in burn wound healing is Flightless I (Flii), an actin-remodeling protein that is upregulated in response to burn injury.^157^ Adams et al^157^ found that the degree of Flii expression correlated positively to TGF-β1 expression and negatively to that of TGF-β3. Through the use of neutralizing antibodies to Flii, partial-thickness burn wound healing in mice was found to be accelerated with decreased remaining wound area when evaluated on day 14 posttreatment.^157^ This was also associated with markedly reduced TGF-β1 and elevated TGF-β3 expression, suggesting that these factors may contribute to the improved wound healing seen with Flii attenuation.^157^

In a multicenter, randomized, double-blinded, stratified clinical trial, Zhang et al^121^ demonstrated that the topical application of a recombinant human GM-CSF (rhGM-CSF) hydrogel compared to placebo yielded significantly accelerated and enhanced partial-thickness burn wound healing. Assessment of the extent of wound healing showed significant differences between the study groups at 14 (84.55% vs 44.44%), 20 (97.55% vs 80.19%), and 28 (100.00 vs 96.67%) days after treatment.^121^ In addition, the time to complete wound healing was found to be 14.71 versus 20.48 days in the treatment and control groups, respectively.^121^ In a separate clinical trial, Wang et al^158^ demonstrated median wound healing time to be decreased to 17 from 20 days and mean wound healing rate to be higher at days 8, 14, 20, and 28 after treatment with rhGM-CSF versus placebo. Pooling the results of these studies together, rhGM-CSF appears to significantly accelerate deep second-degree burn wound healing at 14, 20, and 28 days postburn.^103^

A number of combination growth factor and cytokine agents have been examined in this setting. In a study of placental extracts and their effects on wound healing, Wu et al^159^ found significantly increased expression of TGF-β1 and bFGF and reduced time to complete wound healing of 30 days compared with 52 and 62 days for untreated and normal saline treated rodents, respectively. Amniotic membrane (AM) products and derivatives have also been studied as wound healing agents. Using human, bovine, and acellular bovine AMs, Park et al^160^ demonstrated significantly accelerated reepithelialization with similar therapeutic efficacy between each AM type by 14 days postinjury (76.85%-78.65% in AM groups vs 17.6% in controls) in a porcine burn wound model. Payne et al^125^ demonstrated that the use of ACCS and AMP cells, as described previously in this article, accelerated reepithelialization in guinea pig models of partial-thickness burn wounds. In all but one ACCS-treated group, there was at least 90% wound reepithelialization by day 7 (vs 20%-40% in non-ACCS treatment groups, *P* < .05).^125^ This benefit was also found to be directly proportionate to the dosing of ACCS as evidenced by improved results with higher frequency ACCS application and the presence of AMP cells as a constant source of ACCS.^125^ Regenerating agents (RGTAs), while not growth factors or cytokines in and of themselves, have been found to accelerate and enhance wound healing.^161^ Engineered as derivatives of dextran, RGTAs mimic heparan sulfate in the extracellular matrix, in turn stabilizing and protecting matrix proteins and heparin-binding growth factors (FGF, VEGF, PDGF, TGF-β).^161^ Zakine et al^161^ demonstrated that the use of RGTAs on deep second-degree burn wounds accelerated reepithelialization, improved neovascularization, and decreased the degree of dermal remodeling in a rodent model. However, the time to complete reepithelialization of 7 days was not different between treatment and control groups, although epidermal maturation appeared more advanced with the use of RGTAs.^161^

## FULL-THICKNESS BURN WOUNDS

As full-thickness burn wounds generally require excision and grafting, the utility of growth factors and cytokines as wound healing agents alone is not substantial. The use of these agents as adjuncts to skin grafting and donor site healing will be discussed later in this article. Nevertheless, a small number of studies have demonstrated the potential value of growth factor therapy in this setting.

In 1995, a comparison study between the effects of several different growth factors on deep partial-thickness and full-thickness burn wounds was reported by Danilenko et al.^162^ Using porcine wound models, the group compared recombinant forms of EGF, TGF-α, PDGF-BB, bFGF, KGF, and neu differentiation factor.^162^ Their evidence suggested that significant benefit for full-thickness wounds was only seen with the combination of rPDGF-BB enhancing extracellular matrix production and granulation tissue formation and rKGF increasing epithelialized area.^162^ For this same reason, rKGF was the only agent shown to consistently improve deep partial-thickness burn wound healing.^162^

Jeschke et al^34^ demonstrated that KGF delivered through a nonviral liposomal cDNA gene transfer mechanism significantly increased skin cell proliferation and decreased apoptosis, improved epidermal and dermal regeneration, and enhanced neovascularization compared to controls given only the delivery vehicle (liposomes plus LacZ gene construct) in a rodent model. This benefit was further supported in a similar study by Pereira et al.^163^ Liposomal KGF-cDNA gene transfer in a rodent scald burn model was found to improve reepithelialization, dermal regeneration, and neovascularization through increased expression of IGF-I, IGF binding protein-3, FGF, and collagen type IV and decreased TGF-β production.^163^ Through extensive experience by the group of Herndon and Jeschke, the use of liposomal IGF-1-cDNA gene transfer has been demonstrated to consistently and significantly increase keratinocyte proliferation, accelerate reepithelialization and dermal regeneration, increase serum and liver protein concentrations, and improve postinjury weight gain in rodent models.^141^^,^^164^^-^168 This improvement was also seen to have a positive correlation with increasing numbers of IGF-1-cDNA-containing liposome injections.^164^ These benefits of IGF-1-cDNA gene transfer were found to be associated with increased expression of IGF-I, FGF, KGF, VEGF, PDGF, and collagen type IV.^169^ Jeschke and Herndon^142^ further demonstrated the additional benefit of combining KGF-cDNA and IGF-1-cDNA in liposomal gene transfer constructs over either gene alone in a rodent model. Dermal and epidermal regeneration were significantly enhanced and associated with increases in VEGF expression and neovascularization.^142^

Similar to the study on partial-thickness burns by Singer et al,^91^ TGF-β peptagonist treatment of full-thickness injuries was found to accelerate reepithelialization and reduce scarring and contracture.^99^ Specifically, Huang et al^99^ demonstrated complete versus 70% reepithelialization at day 26 and 50% versus 70% wound contraction at day 33 in treatment and control groups, respectively. Scarring and visually apparent open wound areas were also notably reduced with TGF-β peptagonist therapy.^99^

In the only human study of full-thickness burn wounds found in the literature, Weng et al^170^ reported significantly faster wound healing and lower infection rates with the use of nanometer silver dressing and recombinant bovine FGF compared with saline and paraffin absorbent gauze.

## INTERSTICES OF MESHED SKIN GRAFTS

The goal of treatment for any burn injury is to achieve successful closure of the burn wound. For deeper burns requiring excision and affecting greater amounts of body surface area, the use of meshed skin autografts has helped to overcome the problem of limited donor site availability. However, meshed skin autografts do not impart immediate biologic wound closure as their interstices remain open and expose the patient to metabolic, infectious, and other complications of open burn wounds. The use of growth factor therapy in accelerating the closure of meshed skin graft interstices would have significant therapeutic benefit in this regard. In addition, functional and aesthetic improvements in healed skin graft quality may be secondary benefits of using these agents.

Smith et al^171^ investigated the effect of several growth factors and cytokines in the closure of human meshed skin graft interstices on a “nude” rat model. Accelerated epithelialization and closure of graft interstices were demonstrated in wounds treated with KGF-2, TGF-β2, and bFGF but not interleukin-4, macrophage colony-stimulating factor, or KGF-1.^171^ By day 7, greater than 85% of graft interstices had closed with KGF-2, TGF-β2, and bFGF treatment compared with 65% in controls. While complete closure was reached in untreated controls and all treatment groups (except KGF-1) by day 11, this was achieved in wounds treated with KGF-2 and TGF-β2 by day 9 postgrafting.^171^

The use of ACCS, a solution of multiple growth factors and cytokines as characterized earlier, was demonstrated to accelerate closure of human meshed skin graft interstices by Uberti et al.^130^ By day 9 postgrafting, 83.72% to 92.2% versus 67.03% (*P* < .05) of interstices were epithelialized in ACCS-treated and saline-treated grafts, respectively.^130^ There was no statistical difference seen between daily and alternate day dosing of ACCS or if animal-derived components were a part of the formulation.^130^

Recently, Branski et al^172^ demonstrated that the delivery of PDGF-cDNA via liposomal gene transfer was effective in accelerating reepithelialization and adhesion of meshed skin grafts in a porcine burn wound model. In addition to reinforcing the value of PDGF in the setting, this study further supports the possible use of liposomal gene transfer as an effective mechanism for wound healing therapy in humans.^172^

In 2 separate studies, Akita et al^173^^,^^174^ demonstrated that exogenous bFGF application improved the quality of and color uniformity in skin graft healing. Skin grafts treated with bFGF were found to have accelerated wound healing, better color uniformity with surrounding skin on color meter analysis, and significantly decreased hardness based on a clinical assessment-scoring system used in scleroderma and durometer measurements.^173^^,^^174^

Evidence from these studies suggests that certain growth factors, namely bFGF, TGF-β2, KGF-2, and ACCS, can accelerate epithelialization and closure of meshed skin graft interstices in burn wounds. Improved skin graft healing and survival have been demonstrated in studies on other wound models. Soler et al^33^ showed accelerated closure of graft interstices on a rodent full-thickness excisional wound model with KGF-2 therapy. Vascular endothelial growth factor was found to significantly decrease dermal necrosis and improve survival of full-thickness skin grafts on rodent models in 2 separate, respective studies.^175^^,^^176^ Aside from meshed skin grafts, burn wounds that require more extensive coverage have also been addressed with the use of CSS, which are biopolymer matrices composed of keratinocytes and fibroblasts.^177^ The use of VEGF promoted angiogenesis and organization of murine but not human endothelial cells in CSS grafted to an athymic mouse model, suggesting that it plays a limited role on human endothelial cells and CSS with neovascularization.^178^ In another study, the addition of bFGF to CSS grafted on immunodeficient mice resulted in significantly enhanced angiogenesis and fibroblast proliferation.^177^ Taken together, growth factor therapy may not only accelerate closure of meshed skin graft interstices but also enhance skin graft survival.

## DONOR SITE WOUNDS

The requirement for skin grafting inevitably creates another wound to address, the donor site wound. Conservative medical management generally proceeds without consequence. However, accelerating and enhancing donor site wound healing are potentially beneficial for reducing continued wound care needs and patient burden and in the setting of multiple skin graftings and limited donor site availability. In the wound fluid from split-thickness skin graft donor sites of 24 patients, Ono et al^10^ demonstrated the consistent presence of large amounts of PDGF, interleukin-6, TGF-α, and TGF-β and possibly small quanitities of EGF and bFGF. Therefore, it stands to reason that the use of exogenous growth factor therapy can be of value in the setting of donor site wound healing.

In 1989, Brown et al^41^ examined the use of EGF with silver sulfadiazene compared to silver sulfadiazene alone in the treatment of split-thickness skin graft (0.015 in) donor site wounds in 12 patients. The average time to 25% to 50% and 75% to 100% reepithelialization with adjunctive EGF was decreased by approximately 1 day and 1.5 days, respectively.^41^ Guo et al^179^ also found that using recombinant human EGF in dressing donor site wounds accelerated reepithelialization and decreased wound healing time.

The benefit of FGF on donor site wound healing has been demonstrated in 2 randomized clinical trials. Ma et al^31^ found that topical recombinant aFGF decreased the mean healing time of donor site wounds to 13.07 from 14.71 days (*P* < .001). Recombinant bovine bFGF was shown by Fu et al^28^ to accelerate healing of split-thickness skin graft (0.008-0.01 in) donor site wounds from 14.74 to 10.68 days compared to placebo. However, an earlier clinical study by Greenhalgh et al^180^ did not find any improvement in the rate of epithelialization or time to complete closure (12.9 vs 12.2 days in bFGF- and placebo-treated wounds, respectively) with the use of topical recombinant human bFGF on partial-thickness skin graft donor sites in children.

Herndon et al^60^ examined the effects of recombinant human growth hormone (rhGH) in patients with greater than 40% total body surface area (TBSA) or 20% TBSA full-thickness scald or flame burns. No significant differences in donor site healing were found between subcutaneously administered placebo and rhGH at 0.1 mg/kg/d.^60^ However, when rhGH was administered intramuscularly at 0.2 mg/kg/d, donor site healing time and overall length of stay were decreased by 2 to 4 days and 0.8 days per percent TBSA burn, respectively.^60^ In a subsequent trial by the same group, the effects of rhGH at 0.2 mg/kg/d administered subcutaneously in patients with greater than 40% TBSA full-thickness burns was studied.^181^ With rhGH treatment compared to placebo, coverage of the dermal-epidermal junction by the basal lamina was increased to 68% from 26% at 7 days and mean healing time was decreased to 5.6 days from 7.7 days (*P* < .05).^181^ Associated increases in systemic IGF-1 levels and local expression of IGF-1 receptors, laminin, collagen types IV and VII, and cytokeratin-14 were demonstrated and postulated to play direct roles in the enhancement of donor site healing seen with GH administration.^60^^,^^181^ Another clinical trial by Gilpin et al^61^ demonstrated significant benefit, using 2 forms of rhGH in accelerating donor site healing to 6.0 days with Protropin (Genentech, Inc, San Francisco, California) and 6.8 days with Nutropin (Genentech, Inc) from 8.5 days with placebo (*P* < .01). While increased fibrosis and abnormal scarring have been associated with GH therapy in experimental studies, de Oliveira et al^182^ and Barret et al^183^ did not find any significant adverse effects of rhGH on human scar formation. In addition, Ramirez et al^184^ demonstrated rhGH therapy to be safe and efficacious in the treatment of severely burned children without significant associated increases in morbidity and mortality.

Bergmann et al^128^ did not find significant differences in the appearance or healing time of partial-thickness donor site wounds treated with ACCS in diabetic and nondiabetic pigs. The benefit of ACCS treatment was seen on a microscopic level with a significantly thicker epidermis and more cell layers and rete ridges in diabetic pigs whereas only more rete ridges were seen in nondiabetic pigs.^128^

Evidence from the clinical trials described supports the use of growth factor therapy in donor site wound healing. Taking into consideration the experimental success and potential benefit, further clinical trials are warranted to establish the role of exogenous growth factors in this setting. While combination growth factor therapy may have a role in donor site wound healing, there is no strong evidence in the literature at this time.

## CONCLUSION

Cytokine growth factors, including PDGF, FGB, EGF, TGF-α, VEGF, IGF-I, NGF, TGF-β, GM-CSF, and ACCS, appear to accelerate and enhance wound healing through their physiologic actions and influences. As such, growth factors represent a promising treatment to affect more rapid and effective closure of burn wounds and have shown positive results in many instances experimentally. Although extensive human clinical data are lacking, a number of studies demonstrate encouraging evidence with growth factor therapy in accelerating and enhancing burn wound healing. Further clinical trials are warranted and considerations for sequential growth factor application and/or growth factor cocktails may prove to be more efficacious than the application of a single growth factor.
